# Supplementary material for: A strawberry accession with elevated methyl anthranilate fruit concentration is naturally resistant to the pest fly Drosophila suzukii
Source: PLoS One. 2020 Jun 2;15(6):e0234040. doi: 10.1371/journal.pone.0234040 (PMC7266294; doi:10.1371/journal.pone.0234040)
Supplement: S1 Table — (PDF) [file pone.0234040.s001.pdf]

Table S1. Sample weights, solvent amounts and extraction yields for fractionation.

		Extraction solvent								
sample	Weight	EtOAc			MeOH/H_2O			H_2O		
#2	12.40 g	40 mL	40 mL	40 mL	40 mL	40 mL	40 mL	40 mL	30 mL	30 mL
		yield: 0.20 g			yield: 1.41 g			yield: 0.15 g		
#4	16.43 g	40 mL	40 mL	40 mL	40 mL	40 mL	40 mL	40 mL	20 mL	20 mL
		yield: 0.40 g			yield: 1.34 g			yield: 0.12 g		
#5	16.26 g	40 mL	40 mL	40 mL	40 mL	40 mL	40 mL	40 mL	40 mL	20 mL
		yield: 0.16 g			yield: 1.07 g			yield: 0.12 g		
#6	15.56 g	40 mL	40 mL	40 mL	40 mL	40 mL	40 mL	40 mL	20 mL	20 mL
		yield: 0.12 g			yield: 1.10 g			yield: 0.10 g		
#13	4.73 g	20 mL	30 mL	20 mL	20 mL	20 mL	20 mL	20 mL	20 mL	20 mL
		yield: n.d.			yield: 1.26 g			yield: 0.05 g		
