# Supplementary material for: A strawberry accession with elevated methyl anthranilate fruit concentration is naturally resistant to the pest fly Drosophila suzukii
Source: PLoS One. 2020 Jun 2;15(6):e0234040. doi: 10.1371/journal.pone.0234040 (PMC7266294; doi:10.1371/journal.pone.0234040)
Supplement: Attachment — Submitted filename: 2020-05-04 Response to Reviewers.docx [file pone.0234040.s002.docx]

**Point-by point response**

**Response to the editor and the publisher**

1. Please ensure that your manuscript meets PLOS ONE's style requirements, including those for file naming. The PLOS ONE style templates can be found at http://www.plosone.org/attachments/PLOSOne_formatting_sample_main_body.pdf and <http://www.plosone.org/attachments/PLOSOne_formatting_sample_title_authors_affiliations.pdf>

2. Thank you for stating the following in the Acknowledgments Section of your manuscript:

"The present work has been financed with funding from the Ludwig-Maximilians University of Munich (NG and MP)."

We note that you have provided funding information that is not currently declared in your Funding Statement. However, funding information should not appear in the Acknowledgments section or other areas of your manuscript. We will only publish funding information present in the Funding Statement section of the online submission form.

Please remove any funding-related text from the manuscript and let us know how you would like to update your Funding Statement. Currently, your Funding Statement reads as follows:

"The authors received no specific funding for this work"

3. Thank you for stating the following in the Competing Interests section:

"The authors have declared that no competing interests exist."

We note that one or more of the authors are employed by a commercial company: Hansabred GmbH & Co.

3.1. Please provide an amended Funding Statement declaring this commercial affiliation, as well as a statement regarding the Role of Funders in your study. If the funding organization did not play a role in the study design, data collection and analysis, decision to publish, or preparation of the manuscript and only provided financial support in the form of authors' salaries and/or research materials, please review your statements relating to the author contributions, and ensure you have specifically and accurately indicated the role(s) that these authors had in your study. You can update author roles in the Author Contributions section of the online submission form.

Please also include the following statement within your amended Funding Statement.

“The funder provided support in the form of salaries for authors [insert relevant initials], but did not have any additional role in the study design, data collection and analysis, decision to publish, or preparation of the manuscript. The specific roles of these authors are articulated in the ‘author contributions’ section.”

If your commercial affiliation did play a role in your study, please state and explain this role within your updated Funding Statement.

In response to points 2. and 3.1, we have removed the acknowledgement section and therefore the reference to funding in the text.

We would like to update our Funding Statement as follows:

"The present work has been financed with funding from the Ludwig-Maximilians University of Munich (NG and MP). KO is an employee of Hansabred GmbH. The funder provided support in the form of salaries for author KO, but did not have any additional role in the study design, data collection and analysis, decision to publish, or preparation of the manuscript. The specific roles of this author are articulated in the ‘author contributions’ section."

3.2. Please also provide an updated Competing Interests Statement declaring this commercial affiliation along with any other relevant declarations relating to employment, consultancy, patents, products in development, or marketed products, etc.

Within your Competing Interests Statement, please confirm that this commercial affiliation does not alter your adherence to all PLOS ONE policies on sharing data and materials by including the following statement: "This does not alter our adherence to  PLOS ONE policies on sharing data and materials.” (as detailed online in our guide for authors [http://journals.plos.org/plosone/s/competing-interests](about:blank)).  If this adherence statement is not accurate and  there are restrictions on sharing of data and/or materials, please state these. Please note that we cannot proceed with consideration of your article until this information has been declared.

Please respond by return email with an updated Funding Statement and Competing Interests Statement and we will change the online submission form on your behalf.

Please know it is PLOS ONE policy for corresponding authors to declare, on behalf of all authors, all potential competing interests for the purposes of transparency. PLOS defines a competing interest as anything that interferes with, or could reasonably be perceived as interfering with, the full and objective presentation, peer review, editorial decision-making, or publication of research or non-research articles submitted to one of the journals. Competing interests can be financial or non-financial, professional, or personal. Competing interests can arise in relationship to an organization or another person. Please follow this link to our website for more details on competing interests: [http://journals.plos.org/plosone/s/competing-interests](about:blank)

We would like to update our Competing Interests Statement as follows:

The authors declare no competing interest. The affiliation of KO to Hansabred GmbH does not alter our adherence to PLOS ONE policies on sharing data and materials.”

4. We noted in your submission details that a portion of your manuscript may have been presented or published elsewhere.

"Figure 1A is adapted from one of our recent papers, as explicitely indicated in the text. It is important to show this panel in this context again, to compare it to the next 2 panels. The work is published in open access: https://www.frontiersin.org/articles/10.3389/fpls.2016.01880/full"

Please clarify whether this [conference proceeding or publication] was peer-reviewed and formally published. If this work was previously peer-reviewed and published, in the cover letter please provide the reason that this work does not constitute dual publication and should be included in the current manuscript.

The graphs shown in Figure 1a of this manuscript were previously peer reviewed and published in: Gong, X., et al. (2016). "Strawberry Accessions with Reduced Drosophila suzukii Emergence From Fruits." Frontiers in Plant Science 7(1880), Figure 2b.

The work presented in the present manuscript is the direct follow-up of this former publication. We do think it is valuable to present the relevant data from the previous publication to the reader in the form of Figure 1a. Doing so creates the context of our methyl anthranilate measurements, plotted in Figure 1b,c, which were not previously published. Finally, this composition of Figure 1 allows the reader to directly cross-reference individual data points for fly emergence and methyl anthranilate content.

5. Please include captions for your Supporting Information files at the end of your manuscript, and update any in-text citations to match accordingly. Please see our Supporting Information guidelines for more information: [http://journals.plos.org/plosone/s/supporting-information](about:blank).  [Note: HTML markup is below. Please do not edit.]  Reviewers' comments:  Reviewer's Responses to Questions

We have added the following caption to the manuscript:

**S1 Table.** Sample weights, solvent amounts and extraction yields for fractionation.

**Response to the reviewers**

**Reviewer #1:** Bräcker et al. A strawberry accession with elevated methyl anthranilate fruit concentration is naturally resistant to the pest fly Drosophila suzukii  PLOS ONE 3-28-20  The biological problem in this manuscript has agricultural and economical relevance. This problem is to understand the chemical basis of natural resistance to the crop pest D. suzukii. The authors find that one of the most resistant accessions of strawberries is abundant with methyl anthranilate. Methyl anthranilate alone or fractionated from the resistant strawberry accession decrease the survival of D. suzukii embryos. In contrast to its lethality on their embryos, methyl anthranilate attracts D. suzukii flies, which also prefer to lay eggs on substrates inoculated with this compound. This manuscript is well written and technically correct. Here are some minor comments:

1. The effect of this compound on adult survival requires investigation, especially all the behavior experiments were performed by adult flies. This isn’t necessarily for this study

We do agree that this is an interesting aspect of the relationship of methyl anthranilate and the ecology of *Drosophila suzukii* as a whole. While beyond the scope of this study, measuring survival of adults on various concentrations of methyl anthranilate might reveal new aspects of this compound when interaction with various life stages of this species.

2- In figure 2 caption isoamyl acetate is one word (isoamylacetate), please correct.

Corrected!

3. More concentrations are required to be tested for the oviposition experiments, try to be consistent with the concentrations used for the survival experiments.

We do agree with this comment and initially we probed other concentrations in the oviposition assay. However, we opted to finalize the data on two concentrations. These two concentrations resulted in robust egg laying attraction or aversion, and thus represent the general finding that both preferences for methyl anthranilate can exist in *D. suzukii* females. Furthermore, we do think that both concentrations are close to the operating maximum/minimum of the assay. For example, higher concentrations of methyl anthranilate will flood the limited volume of the egg laying cage with the olfactory stimulus, making it harder to establish a stimulus difference between both sides which is necessary to detect the choice behavior.

4-. What is the amount of the purée in the egg-laying preference assay?

Each plate was filled with 20 ml of purée in total (10 ml on each half). Purée was prepared in large 200 ml batches to facilitate dilutions and minimize variance between experimental days. We added this information to the methods section.

5- the same for the trap assay experiments, try to be consistent with the concentrations used for the survival experiments.

Based on the difference between both assays, with one being an ovipositioning assay in which the flies are directly exposed to the medium, and the other being a trap assay that only allows olfactory stimulation via longer distances, we do not think that methyl anthranilate concentrations are comparable in a precise one to one relationship. Furthermore, the trap assay aims to isolate the reaction to the compound and thus uses only water to dilute. We thus opted to test a range of concentrations in both assays, demonstrating that the general observation of attraction and aversion to methyl anthranilate can indeed be made in both assays.

**Reviewer #2**: The authors found that a particularly resistant strawberry accession to Drosophila suzukii had a high concentration of methyl anthranilate (MA). Through several accurate bioassays including different concentrations, the authors demonstrated that MA is indeed toxic to the embryos, in spite of its olfactory attraction to the females and their preference to lay eggs on media with low toxic concentrations. This result is very interesting and very promising for agriculturalists as well as scientists interested in the biology of insect-plant interactions. While I recommend the acceptance of the paper as it is, I believe that two additional points may be raised or discussed by the authors. The first point considers the relative significance of MA in strawberry resistance. The authors noted that the MA-based resistance is unique to a single accession indicating that variable resistance mechanisms may exist in different strawberry cultivars and species. In a previous paper, they showed that emergence was correlated with fruit size. It might hence have been preferable to show on a graph whether the resistant accession P300 has also a smaller size (e.g., a 3D plot with MA concentration, fruit size and average emergence per strain/species). The authors had also measured the concentration of other substrates for each strain/species. A multivariate regression analysis of average emergence over the different chemical concentrations, water loss (if measured for all strains/species) and fruit size could also be helpful to explain how much MA could explain fruit resistance.

We did consider a multivariate regression analysis of average emergence over different parameters when preparing this manuscript. We did not do it in the end for the following reason. The emergence is a variable trait where the average is not representative enough. Moreover, it is technically not possible to correlate the emergence from individual fruit to the metabolite data, which is an average measured from a batch of fruits. What would be meaningful, but at the moment not doable, is to repeat the emergence assays and the metabolite measurements on the same individual fruits (e.g., on fruit halves), to then carry the kind of multivariate analysis suggested by the reviewer.

In our former paper (Gong et al., 2016), we did show that emergence was best explained by genotype and fruit size across the 107 strawberry accessions we tested. Some accessions, however, departed from this trend and showed less emergence than what fruit size would predict. This was the case for accession 300 for which the higher resistance might be explained by methyl anthranilate concentration. We have added a sentence in the introduction to clarify and underscore this point.

Finally, we want to stress that, although we cannot currently evaluate the specific contribution of methyl anthranilate to the resistance, relative to other factors, we did confirm that methyl anthranilate concentration in 300, measured in different batches of fruits, grown in different years was stable and was always the highest of all accessions tested. Likewise, we have repeated the emergence assays on accession 300 in 2015, 2016, 2017, 2018 and 2019 and found the emergence rate to be very consistently low.

The second point considers the significance for MA adaptation in D. suzukii. The authors compared this situation (a female preference to a toxic substance) with D. sechellia adaptation to Morinda citrifolia. However, D. sechellia is an island species and it might have been obliged to adapt to its host. For D. suzukii, the geographical distribution and the niches are far broader. High MA concentration may not be as frequently encountered to necessitate particular genetic adaptation, either in native or introduced populations.

We do agree with this remark. By comparing our findings to the behavior of *D.sechellia*, we intended to show the existence of other seemingly contradictory preference behaviors in the context of oviposition. We added to this section, to ensure that the reader understands it as a general comparison.
